# Barriers on the pathway to survival for children dying from treatable illnesses in Inhambane province, Mozambique

**DOI:** 10.7189/jogh.09.010809

**Published:** 2019-06

**Authors:** Karin Källander, Helen Counihan, Teresa Cerveau, Francisco Mbofana

**Affiliations:** 1 Malaria Consortium London, UK; 2 Karolinska Institutet, Stockholm, Sweden; 3 Malaria Consortium Mozambique, Maputo, Mozambique; 4 Ministerio de Saúde, Maputo, Mozambique

## Abstract

**Background:**

Mozambique has one of the highest under-5 mortality rates in the world. Community health workers (CHWs) are deployed to increase access to care; in Mozambique they are known as agentes polivalentes elementares (APEs). This study aimed to investigate child deaths in an area served by APEs by analysing the causes, care seeking patterns, and the influence of social capital.

**Methods:**

Caregivers of children under-5 who died in 2015 in Inhambane province, Mozambique, were interviewed using Verbal Autopsy/Social Autopsy (VA/SA) tools with a social capital module. VA data were analysed using the WHO InterVA analytical tool to determine cause of death. SA was analysed using the INDEPTH SA framework for illnesses lasting no more than three weeks. Social capital scores were calculated.

**Results:**

117 child deaths were reported; VA/SA was conducted for 115. Eighty-five had died from an acute illness lasting no more than three weeks, which in most cases could have been treated at community level; 50.6% died from malaria, 11.8% from HIV/AIDS, and 9.4% for each of diarrhoea and acute respiratory infections. In 35.3% the caregiver only noticed that the child was sick when symptoms of very severe illness developed. One in four children were never taken outside the home before dying. Sixteen children were first taken to an APE; of these 7 had signs of very severe illness. Caregivers who waited to seek care until the illness was very severe had a lower social capital score. The mean travel time to go to the APE was 2hrs 50min, which was not different from any other provider. Most received treatment from the APE, 3 were referred. The majority went to another provider after the APE; most to a health centre.

**Conclusions:**

The leading causes of death in children under-5 can be detected, treated or referred by APEs. Major care seeking delays took place in the home, largely due to lack of early disease recognition and late decision-making. Low social capital, distance to APEs and to referral facilities likely contribute to these delays. Increasing caregiver illness awareness is urgently needed, as well as stronger referral linkages. A review of the geographical coverage and scope of work of APEs should be conducted.

The last two decades have seen impressive progress in global health. There were 7.2 million fewer deaths globally of children under five in 2017 than there were in 2000, with half of the lives saved attributed to the gains made in preventing and treating pneumonia, diarrhoea, intra-partum related events, malaria and measles [1]. Malaria deaths alone fell by 62 percent between 2000 and 2015, largely as a result of increased efforts and a huge mobilisation of funding [2]. Progress, however, has been uneven, with the poorest and most vulnerable still being left behind, largely due to a lack of access to vital health services [3]. In many rural villages access to health facilities is limited. The majority of the 5.6 million children who die each year still die from illnesses that are easily preventable and treatable [4]. This is exacerbated by a global health worker shortage of over seven million – a figure which is expected to rise to nearly 13 million by 2035 [3,5]. As the international community mobilises towards realising the Global Goals for Sustainable Development, it is clear that more needs to be done in order to achieve universal health coverage and secure healthy lives for all by 2030.

Community-based primary health care (CBPHC) is a key mechanism to delivering health services to hard-to-reach and under-served communities. CBPHC involves using trained community health workers (CHWs), who may or may not be paid, to deliver health services to these communities. CHWs are provided with training, tools and medicines and supplies to deliver basic health services to the rural communities in which they live. This allows CBPHC programmes to reach into the heart of communities to tackle the major causes of childhood and maternal illness and mortality. In this way, CBPHC leverages greater efficiencies from health services and has the potential to address many of the remaining challenges in global health, becoming an essential vehicle in the achievement of the Global Goal on health.

CHWs have been a foundation for CBPHC in Mozambique since 1978; nationally referred to as *Agentes Polivalentes Elementares* (APEs) [6,7]. The APE programme faced many challenges and was severely disrupted by the 1977-1992 civil war. In particular, the lack of supervision and commodity supply systems resulted in the interruption of programme implementation in the mid-1990s [8]. In 2010, the Ministry of Health (MoH) launched a revitalization programme for the APEs and as of December 2016, 3524 APEs had been trained to conduct health-promotion activities and provide integrated community case management (iCCM) for malaria, pneumonia and diarrhoea for children aged 2-59 months. They also treat all age groups for malaria and diarrhoea, refer acute malnutrition cases, newborns, pregnant women with danger signs and register all births and deaths in their communities. In 2014, the services APEs provide were expanded to include family planning, pregnancy tracking, antenatal care, post-partum care, healthy child check-ups, as well as TB and HIV patient follow-up for treatment adherence counselling [9]. According to the MoH, 80% of the APE workload should be devoted to health promotion activities, including promoting the use of health services, and encouraging caregivers to bring the sick child in a timely manner for appropriate care [10]. Medicines for APEs are provided from the central medical stores (CMAM) through a kit system with funding from the World Bank, Global Fund and the President’s Malaria Initiative. APEs receive a monthly stipend of 20 USD (1200 MZM in 2017). Oversight, training and support to APEs are provided by the province, district and health centre, with a designated APE supervisor at the nearest health centre providing direct supervision to 5-8 APEs.

While under five mortality in Mozambique, estimated at 79 per 1000 live births in 2015, has reduced by more than two-thirds from the rate in 1990, the mortality rate is still among the highest in the world. Due to an inadequate civil registration and vital statistics (CRVS) system, more than 2.5 million children are unregistered and the causes of over 300 000 deaths are unknown in the country [11]. As a result, the modifiable factors at the level of the household, community and health facility that could have prevented these deaths are also unknown. Risk factors for fatal outcomes in sick children include poor socioeconomic status, incomplete immunization schemes, malnutrition, late care seeking and inadequate treatment [4]. Yet cheap and effective tools exist for most childhood infections [12], many which are included in the kit used by the APEs in Mozambique. Lack of social capital, i.e, the (usually non-monetary) resources generated through social networking and involvement in community affairs (eg, sense of belonging, trust and influence) [13], has also shown to be associated with poorer health outcomes and behaviours [14,15]. While social capital as a concept is increasingly being used to help policy-makers and programmers understand how formal and informal networks within and among communities can foster better governance and accountability, as well as contribute to improvements in health, health financing and the equitable delivery of health services [13], it has never been explored in the context of understanding care seeking patterns for child deaths. This study used verbal and social autopsy methodology, combined with a short Social Capital Assessment Tool to investigate the biological causes of child death, care seeking preceding death and the structural (ie, community group membership, emotional/economic support from individuals) and cognitive social capital (ie, trust, social harmony) in an area covered by APEs, to understand why children fall off the pathway to survival.

## METHODS

### Study design

**Figure 1:**
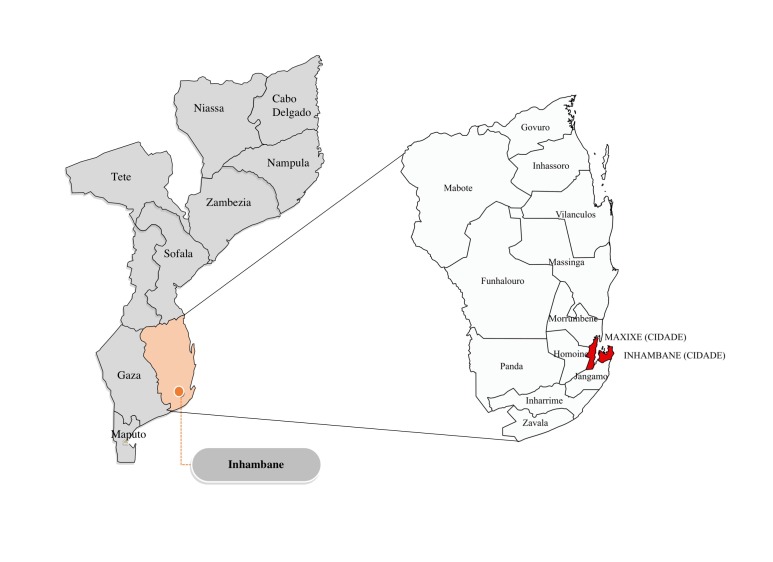
Map of Mozambique, Inhambane province, and its districts. The red areas are the urban district of Maxixe and Inhambane City, which was excluded from the research.

A community-based investigation of child deaths in Inhambane province in Southern Mozambique was conducted. The target population included all families living in any of the non-urban 11 districts where an APE was serving their community (**Figure 1**). The total population of children 2-59 months in areas covered by APEs in Inhambane in 2015 was estimated at 62 475 children [16], with one APE serving a population of 2500-5000.

Before entering into the field, the study team spent time with the APEs and the community leaders explaining the study. Due to the nature of the research, a four-month mourning period was left between the death of the child and the interview. Family members were given enough time to read the participant information sheet and the key points were summarized verbally to all participants who could not read; all questions that arose were answered. Written informed consent of all participants was obtained; for those who could not read or write a fingerprint was requested. Participants were informed that the decision to participate was entirely voluntary and that the respondent could refuse to answer questions without any consequences.

### Data collection

All of the 12 monthly reports submitted by 275 APEs serving 11 of the 12 districts in the province were collected for 1^st^ January to 31^st^ December 2015. From these, information on deaths in children 2-59 months were extracted and with the help of the APE who had submitted the data, together with the community leader, the deaths were verified and the households of these children located. One of the eight research assistants was responsible for investigating the circumstances of the deaths, from initial illness through to death, by interviewing the family to ascertain the sequence of events and their health-seeking behaviour.

A standard WHO verbal autopsy (VA) tool [17], the INDEPTH network (http://www.indepth-network.org/) social autopsy (SA) tool [18,19] and a simplified short social capital tool adapted from the World Bank [20] were programmed in CommCare [21] into a tablet based interview tool in Portuguese, which was used to interview caregivers of children who had died. The VA tool is designed to help determine probable causes of death in cases where there was no medical record or formal medical attention given, whereas the SA tool is designed to investigate the socio-cultural, behavioural and health systems factors that contribute to child deaths. The social capital tool was designed to create an understanding of the networks of relationships among people who live and work in a particular society, as low social capital has shown to have a negative impact on health outcomes [22]. For example, “groups and networks” included questions on the household member's participation in various types of social organisations; for “trust and vulnerability”, questions considered if, in general, people can be trusted and if people can get emotionally or morally hurt; and for “collective action and cooperation”, questions were asked about how members of a community supported each other in response to a crisis. It is important to note that no validated social capital tool existed at the time of this study and our questionnaire was drawn up from a combination of previous studies on antiretroviral therapy adherence [23] and from an integrated questionnaire for measurement of social capital by the World Bank [19].

The data collectors were hired at provincial level and conducted data collection under the supervision of the National Institute of Health (INS), the Ministry of Health APE Programme Manager, the Provincial Health Directorate (DPS), and Malaria Consortium. The seven-day training of the data collectors covered instructions on survey implementation, the use of tablets (Lenovo Tab4 8”; Lenovo, Morrisville, NC, United States) for data collection, the need for confidentiality and privacy, and interviewing techniques, and involved a combination of classroom teaching and role plays, followed by two days of field testing. The interview tools were in Portuguese, but interviewers were trained to do on-the-spot translation of questions into the local languages of the communities (Changana, Nyanja and Makhuwa). There was structured regular supervision in the field throughout the data collection period, and all questionnaires completed using the tablets were reviewed after the interview by the interviewers themselves and subsequently by the field supervisors on a daily basis. Checks and rules were built into the application to reduce missing data or data entry errors.

### Data analysis

The CommCare application synchronised data with a server from which a data set was downloaded into Excel. The VA data were exported and integrated into the WHO InterVA**-**4 (v4.04) analytical tool to determine likely cause of death using the built in probabilistic models [24]. For those with an acute illness (<3 weeks) the SA data were analysed in STATA 13 (StataCorp, College Station, TX, US) and Microsoft Excel (Microsoft Inc, Seattle WA, USA), using the INDEPTH SA framework [19] to analyse common bottlenecks and delays to appropriate care.

Social capital was measured using two dimensions, namely structural and cognitive, and within those there were four different categories:

1. Number of groups: the number of groups or organisations to which the participant belonged (e.g. religious group, political club, etc.). One point was allocated per group, with a maximum score of four.
2. Trust: if the participant had trust in people in general. A score of zero was allocated for a negative response; five points was allocated for a positive response.
3. Vulnerability: If the participant felt that people in general can get hurt. A score of five was allocated for a negative response; zero points was allocated for a positive response.
4. Ability to borrow money from someone, including friends and relatives. Points were allocated from one to five, based on a Lickert scale (from ‘certainly not’ to ‘certainly’).

All the statistical analysis was descriptive: confidence intervals are reported where appropriate, on the basis of the standard error of a proportion. The main analysis included a description of the steps in the pathway to care seeking preceding the death, as well as the “three delays analytical model”, as suggested in the literature [19,25,26]. An absolute total social capital score was calculated similar to the method by Kang et al [14], based on the responses to the questions listed above. The lowest possible score was 0 and the highest was 19. Bivariate regression using Student’s *t* test was used to document the association between mean social capital score and key steps in the social autopsy pathways to care seeking.

### Ethical considerations

Ethical approval was obtained by the bioethics committee of the National Institute of Health (CIBS-INS) in Maputo, Mozambique in October 2014 (356/CIBS-INS/2014 and 236/CNBS/2015). The survey was conducted in accordance with the principles of good practices and all information collected during the study was treated with strictest confidence. Personal data, particularly name, geographic information and contacts that were used to localise the household of the deceased, were kept separately from the data collection forms used by the research assistants, to protect privacy and ensure confidentiality.

## RESULTS

### Background characteristics

A total of 117 child deaths were extracted from the APE monthly reports, of which VA/SA were conducted for 115. Of these, 85 had died from an acute illness that had lasted less than three weeks and these were the ones included in the analysis. The majority (91.8%) of the deaths occurred at home (80.0%) or on the way to hospital (11.8%). Of the 85 deaths investigated, 52.9% were girls and 88.2% (75/85) were reported to normally sleep under a bed net. Half of the deaths occurred in children 12-59 months old (43/85; 50.6%), whereas 49.4% (42/85) occurred in infants (1-11 months). Of the 85 deaths investigated, 72 (84.5%) had information available for a social capital score (13 responded “don’t know” to at least one of the questions). The mean total social capital score was 11.2 (range 1-19).

### Cause of death

Malaria was the main cause of death (COD), accounting for 50.6% of deaths (43/85), followed by deaths related to HIV/AIDS (10/85; 11.8%), diarrhoeal diseases and acute respiratory infections (both 8/85; 9.4%). Nine children were also assigned a second possible COD, with meningitis/encephalitis (3/9) and malaria (2/9) being the main possible secondary causes (**Table 1**).

**Table 1:** Causes of death in children under five (n = 85)

1^st^ cause of death	No. (N = 85)	%
Malaria	43	50.6
HIV/AIDS* related death	10	11.8
Acute respiratory infection (including pneumonia)	8	9.4
Diarrhoeal disease	8	9.4
Epilepsy	5	5.9
Acute abdomen	3	3.5
Undetermined	3	3.5
Asthma	2	2.4
Congenital malformation	2	2.4
Sepsis (non-obstetric)	1	1.2
**2^nd^ cause of death**		
Meningitis and encephalitis	3	33.3
Malaria	2	22.2
Acute respiratory infection (including pneumonia)	1	11.1
HIV/AIDS* related death	1	11.1
Diarrhoeal disease	1	11.1
Acute abdomen	1	11.1

*Human immunodeficiency virus/Acquired immunodeficiency syndrome

### Care seeking pathways preceding death

**Figure 2:**
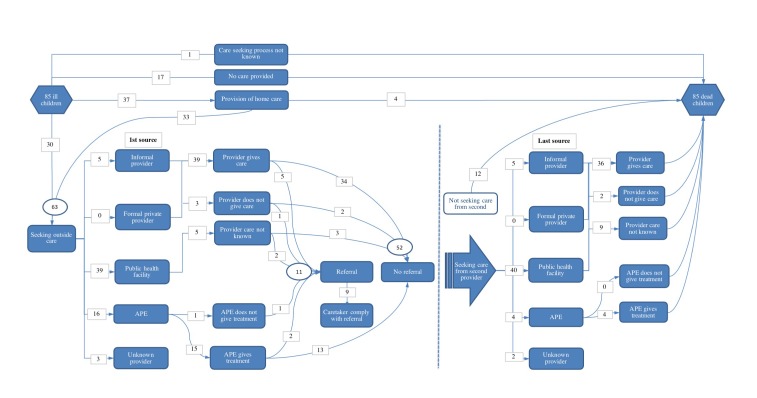
Pathway of care seeking for 85 child deaths, showing the providers and provider actions for the first and last source of care sought.

**Figure 2** shows the pathways for care seeking that children followed before dying. In 36% (31/82; 3 did not know the status) of children the caregiver only noticed that the child was sick when symptoms of very severe illness had developed (stopped eating, unconscious or stopped moving). The majority (67/85; 78.8%) had received some form of treatment before dying, whereof 55.2% (37/67) were first treated with mainly herbal medicines at home before seeking outside care (25/37; 67.6%). 75% (63/85) were taken outside the home for care before death; of the 21 (25.0%) children who were never taken outside the home, most of these (17/21) had not received any treatment before death. When asked why they had not sought any care for these children, caregivers answered that the child died suddenly before they were able to initiate care seeking (7/21; 33.3%), because they did not realise that the illness was serious (4/21; 19%), and various other reasons (10/21; 47.6%) (used medicines /herbs they had at home, parents were not at home, had no transport/registration card/money, thought the illness was related to a recent vaccination, or could not explain why).

Of children who were taken outside the home, 25.4% (16/63) were first taken to an APE, whereas 52.4% (33/63) (were taken to a health facility, 9.5% (6/63) to a hospital, 7.9% (5/63) to a traditional healer (and three were taken to an unspecified location. Of the children who were first taken to an APE, 43.8% (7/16) were reported to have had signs of very severe illness when the caretaker took the decision to seek care, whereas 56.3% (9/16) were moderately ill (ate poorly, sleepy or less active than normal). While the children who were first taken to a health facility were less often severely ill (30.3%) compared to those who first when to an APE, the difference was not significant (*P* = 0.35). None of the children were taken to the APE when they had mild illness (sick but eating normally, being alert and active). The mean time to walk to the APE as first provider was 2hr 50min, which was not significantly different compared to other providers (*P* = 0.72). All except one child received treatment from the APE; seven reportedly received an antimalarial, one paracetamol, and eight did not know the medicine. Three children who first went to an APE were referred to a health facility. There was no significant difference in referral rate between those who first went to an APE vs another provider (*P* = 0.88). The majority (12/16; 75%) of children who were first seen by an APE were taken to a second provider later in the illness; most to a health centre (9/16; 69.2%). Two children were taken back to the APE before dying. The mean time spent to get to the last provider was 3.12 hours (SD 5.84) and 33.9% of caregivers travelled by foot. A total of 16 (25.4%) of the children who sought outside care during the illness were hospitalised; there was no significant difference in hospitalisation rate between those who first went to an APE and those who went to other providers (*P* = 0.64).

### Decision making in the household

Several questions were asked about the decision-making processes in relation to different decisions taken in the household. In general, women (47.1%; 40/85) were significantly more often the ones in charge of decisions relating to routine decisions in the home (buying and selling food, daily activities in the home) than when big decisions were made (moving house, buying/selling or rebuilding the house, renting the land) (10.6%; 9/85) (*P* < 0.0001). For other decisions, such as whether to visit relatives or friends, to take a sick relative to a health facility, or take a sick child to a health facility or APE, on average 49.4% stated that this was decided by both the husband and the wife and none of these decisions were stated to involve women less than the men. On the contrary, more women were solely responsible for the decision to seek care for their sick children that men (22.3% vs 11.6%) (*P* = 0.11) and 96.5% (82/85) responded that do not need to seek permission from their husbands before seeking care from a health facility or APE for their sick child.

### Social capital

Mean social capital scores for each of the pathway indicators are reported in **Table 2**. Out of the four categories included to form the score, the highest principal component eigenvector loadings were found for Vulnerability (0.61) and Trust (0.58), whereas Ability to loan money (0.41) and member of Number of groups (0.36) had less loading. The only indicator that was associated with social capital was the status of the child when the caregiver detected that the child was ill; caregivers who only detected the illness when the child had severe symptoms reported a significantly lower social capital score (*P* = 0.0007).

**Table 2:** Pathway indicators and social capital score*

Indicator	Number of deaths	%	Mean social capital score (SD)	95% CI	P-value
**Child status when caregiver detected that the child was ill:**					
Mildly or moderately sick	46	64.8%	12.6 (3.9)	11.4, 13.7	0.0007
Severely sick	25	35.2%	9 (4.3)	7.2, 10.8	
**Children who were treated at home:**					
No	40	55.6%	11.2 (4.7)	9.7, 12.7	0.94
Yes	32	44.4%	11.3 (4.0)	9.8, 12.7	
**Children who were neither treated in nor outside the home:**					
No	57	80.3%	11.1 (4.2)	10.0, 12.2	0.43
Yes	14	19.7%	12.1 (5.2)	9.1, 15.2	
**Caregivers waiting >1 day to seek care after recognising the symptoms:**					
No	23	31.9%	10.7 (4.1)	8.9, 12.4	0.44
Yes	49	68.1%	11.5 (4.5)	10.2, 21.8	
**Children who were taken outside the home for care:**					
No	17	23.9%	13 (5.2)	10.3, 15.7	0.07
Yes	54	76.1%	10.7 (3.9)	9.7, 11.9	
**Children whose caregiver waited >3 hours to seek care after decision had been taken:**					
No	24	44.4%	11.6 (3.8)	10.0, 13.2	0.16
Yes	30	55.6%	10.1 (4.1)	8.6, 11.6	
**Caregivers who delayed >2 hours to reach first provider:**					
No	44	81.5%	10.8 (3.7)	9.6, 11.9	0.98
Yes	10	18.5%	10.8 (0.3)	7.0, 14.6	
**Children who were taken to an informal first source of care:†**					
No	49	94.2%	10.7 (4.1)	9.5, 11.8	0.58
Yes	3	5.8%	12 (3.5)	3.4, 20.6	
**Children who were taken to an APE as first source of care:†**					
No	40	74.1%	11.0 (4.1)	9.7, 12.3	0.54
Yes	14	25.9%	10.2 (3.7)	8.1, 12.3	
**Cause of death – malaria:**					
No	35	48.6%	11.5 (3.7)	10.2, 12.8	0.60
Yes	37	51.4%	11.0 (5.0)	9.3, 12.6	
**Cause of death – HIV/AIDS:**					
No	64	88.9%	11.3 (4.5)	10.1, 12.4	0.87
Yes	8	11.1%	11.0 (3.9)	7.7, 14.3	
**Cause of death – ARI, including pneumonia**					
No	65	90.3%	11.0 (4.4)	10.0, 12.1	0.26
Yes	7	9.7%	13 (4.3)	9.0, 17.0	
**Cause of death – diarrhoea:**					
No	65	90.3%	11.4 (4.6)	10.2, 12.5	0.38
Yes	7	9.7%	9.9 (1.8)	8.2, 11.5	

ARI – acute respiratory infection, APE – agentes polivalentes elementares

*The total number varies because of missing data (due to “don’t know” answers) in both the pathway indicator and the social capital indicators.

†Out of the ones who were taken outside the home for care.

‡P<

### The three delays

**Figure 3:**
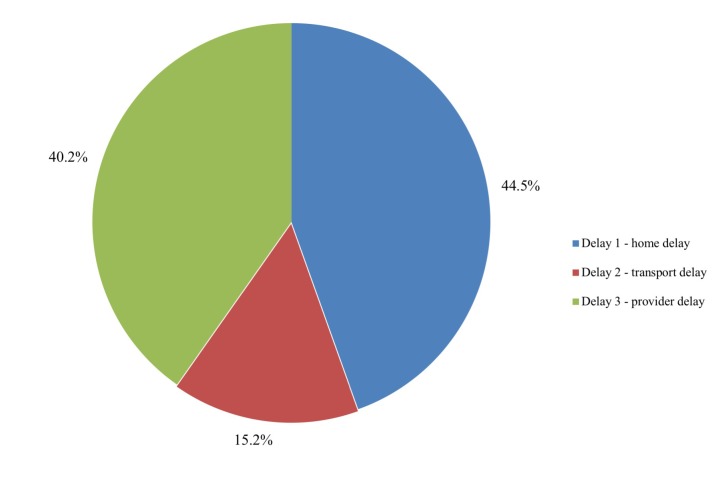
Different types of delay preceding death of children.

The relative contribution of delays at the home, on the way to, or in the health facility was calculated (**Figure 3**). Most delays in the care-seeking process were caused by problems at home (44.5%). The second biggest contribution to delay (40.2%) was caused by problems at the health provider, whereas transport delays contributed to 15.2% of the total delay.

#### Delays in the home

31 (37.8%) of the 82 caregivers who gave information about the child’s state had only realised that the child was ill when the condition was severe, and 21 (24.7%) were not taken out of the house for treatment. Some narratives on the reasons why caregivers did not seek care outside the home, or sought care late are provided in **Box 1**.

Box 1 **Sample of narratives for reasons why caregivers did not seek timely care outside the home**

Caregiver 1: “The child’s parents had not given approval [to take the child for care] as they had already gone to Maputo [the capital of Mozambique].

Caregiver 2: “I lacked immediate transportation, and at the time [of the death] the APE was not available”

Caregiver 3: “When I got home from the field I found my child very ill and she died shortly after.”

Caregiver 4: “I thought it was a simple fever that could resolve by itself.”

Caregiver 5: “We thought that we could wait a little bit and take the child [for care] the next day, but he ended up losing his life that night.”

Caregiver 6: “I thought the child was healthy.”

Caregiver 7: “The mother of the child did not take the child to the health unit because she claimed that they would not see them there because they don’t have a health card”.

Caregiver 8: “Everything happened very fast and the baby died while we were getting ready to go to the hospital.”

Caregiver 9: “Because I had no money to go for care.”

Caregiver 10: “Because he had gone to the hospital on Friday to get the baby vaccinated and I thought the illness was vaccine-induced.”

Of children reported with moderate or severe disease, 34 (47.9%) delayed more than one day before seeking health care and 33 (57.9%) delayed more than three hours to act on the decision to go outside for care; the main reason being that they believed that the child had improved or the symptoms had stopped. Of the 11 children who were referred from at least one provider, only two did not adhere to the referral advice.

#### Delays due to transportation

Of the 63 children taken for care outside the home, 3 (5.3%) delayed more than three hours to act on the decision due to lack of transport, and 13 (21.0%) delayed more than two hours to reach the provider after having found a transport method. The majority (43/63; 68.3%) went by foot to the health provider. Of the two children who failed to adhere to referral neither were mentioned to have had difficulties in finding transport.

#### Provider delay

Most children (93.1%; 54/58 (5 missing data)) who sought care from at least one health provider received some kind of treatment, but 18 (31.0%) of them had to wait more than one hour to receive the treatment. Of the 11 children who were referred, 3 (27.3%) were referred because of lack of medicines or equipment.

The total delay score at home, transport or provider was calculated for each provider first seen, among the children who were taken outside for care before dying (**Table 3**). The home delay was significantly lower among the children who were first taken to an APE compared to other providers (*P* = 0.02) but was significantly higher among the children who were taken to a traditional healer (*P* = 0.04). The children who were taken to a health facility had a significantly higher transport delay score than other providers (*P* = 0.004), and the children who were taken to traditional healers had a significantly higher provider delay score (*P* = 0.04), whereas those who went to health facilities had a lower provider delay score.

**Table 3:** Home, transport and provider delay score by first provider seen (N = 63)

	Home delay score (max = 7) N (95% CI^#^)	*P* value*	Transport delay score (max = 3) N (95% CI)	*P* value	Provider delay score (max = 3) N (95% CI)	*P*-value
APE	2.13 (1.61, 2.64)	0.02	0.13 (0.0, 0.31)	0.24	1.06 (0.93, 1.20)	0.19
Health facility	2.64 (2.34, 2.93)	0.77	0.42 (0.21, 0.64)	0.004	0.76 (0.54, 0.98)	0.03
Hospital	2.83 (1.80-3.87)	0.53	0	0.20	0.83 (0.40, 1.26)	0.75
Traditional healer	3.0 (1.48, 4.52)	0.04	0	0.25	1.4 (0.72, 2.08)	0.04

CI – confidence interval, APE – agentes polivalentes elementares

*Compared to the other three categories combined.

## DISCUSSION

In this study, we found that almost all the illnesses leading to the deaths in children younger than 5 years were preventable and treatable. The majority could also have been detected, treated or referred by community-based providers such as the APEs. Yet, many did not visit the APE during the child’s illness, or reached when it was already too late for the APE to manage the child. The main contributors to death documented in this study included lack of caregiver awareness and recognition of illness symptoms, home care practices leading to delays in outside care seeking, and considerable distances between households and health facilities. While these factors have previously been established as key barriers to care for young children [27,28], several studies have suggested that these access barriers could be overcome through the introduction of integrated community case management (iCCM) delivered by community health workers (CHWs) [26,29]. However, in our study, we show that the same care seeking delays that caregivers experience when seeking care from distant health facilities also apply for families living in communities served by CHWs providing iCCM. The Mozambique APE programme is unique from other community based primary health care (CBPHC) programmes, in the sense that APEs do not have a fixed health post and that 80% of the time they are supposed to be conducting household visits; making it difficult for caregivers to know where and when to access the APE. With so few caregivers seeking care from an APE, and seeking care too late in the illness process, it is likely that APEs are simply not close enough to the households to be able to cover the access gap. The current APE strategy in Mozambique aims to expand the reach of care to the 20% of population who do not have access to a health facility, but as the strategy calls for 25 APEs per districts as a blanket recommendation, this can result in an APE:population ratio of 1:5000 or more, depending on the district. Hence, we recommend that a review of the geographical coverage of APEs, and the time they spend delivering curative services, should be conducted and that more APEs are deployed in areas where access is still problematic. Most delays happened at home, as a result of caregiver decision making processes or failure to recognise illness symptoms before the child was already severely ill (stopped eating/drinking, stopped moving, or unconscious). The lack of caregiver awareness of symptoms of malaria, pneumonia and diarrhoea, and of the potentially life-threatening nature of these conditions in a young child, has been documented previously [30-35]. In this setting, both parents are in charge of the decision to seek care for a sick child. Yet, while women do not need permission from their husbands to seek care, they may not have access to the cash that the care seeking process requires. While it may be logical to a poor and rural family to wait and hope for improvement in a febrile child before deciding to seek outside care, which often requires using sparse household savings, an untreated malaria infection can progress rapidly to convulsions, coma and death within 24 hours of symptom onset [36]. Instead, families have commonly reported resorting to herbal treatment as “first aid” for fever before seeking outside care [35,37], potentially delaying care seeking from a trained health provider. This practice was also common among the families of the deceased children in our study, including the children who were later taken to an APE for care. While household delays in care seeking have been documented for children seeking care from (often distant) health facilities and hospitals, it was unexpected to see that one in four children in our study never even left the home during the illness before they died.

Use of APEs among these children who later died was low, with only one of four first seeking care from their APE, and of these, most came late when the symptoms were already severe. More children with severe conditions were first taken to the APE than the facility, potentially because the APE was nearer than any other provider. None of the children had been taken to the APE at a stage when the illness symptoms were mild and still treatable by the APE with medicines that could be given at home; still, all but one child had received treatment from the APE. The low capacity of CHWs to recognise, manage and refer severe illness in children has been documented previously, with only about one third of children with severe illness being managed correctly and just over half being referred appropriately by health extension workers in Ethiopia [38]. The failure to recognise and act on signs of serious illness is also problematic in official primary care facilities, and a recent study showed that danger signs were missed in 43% of cases in Mali and in 39% in Uganda, and 45%-51% of patients who were seriously ill were not referred to a hospital in time [39]. These failures are in part due to insufficient training, feedback, and support for the staff at work, and it has been suggested that improvement is possible with introduction of better diagnostic tools to diagnose severe illness [40,41], along with local panel meetings to audit and improve health worker practices and work culture with feedback, education, and leadership [39].

The proportion who decided to seek care outside the home when the child was still moderately ill (ie, sick but not yet unmanageable by an APE) was significantly higher among families with a higher social capital score. While our results showed that a few components of social capital (eg, if the participant had trust in people in general and if the participant felt that people in general can get hurt) provided more loading to the principle component, they were not independently associated with the pathway indicators in binary analysis. However, social capital has been found important in promoting health globally, with associated effects on both health outcomes of people living with HIV/AIDS [15] and child nutritional status [14]. According to Ogden et al [13] the process of building social capital can support health policy and health system strengthening by creation of trust, norms of reciprocity, rights and sanctions across the health system and reaching into the community. Social capital can be accrued by groups of like-minded people within a community and is strengthened as those groups connect with other networks in pursuit of common goals. Our results support the call for further studies examining the possible linkage between child health seeking behaviours and social capital components. Depending on the available types and strengths of specific components of social capital (or the lack thereof), programme practitioners may be able to consider tailoring the caregiver-targeted health seeking programmes, eg, community empowerment interventions such as women’s groups [42] or positive deviance [14], to effectively influence related behaviour changes.

The “three-delays” model has been used in previous studies to examine barriers to seeking care and preventing maternal and child mortality [19,26,29,43,44]. Children who die experience major delays of various kinds, largely due to lack of early recognition of the disease in the home and late decision-taking for outside care seeking. Distance to APEs and to referral facilities likely contributes to these delays, both for moderately and severely sick children. In this study, the main delays that prevented the children to receive health care in a timely manner are in line with other studies in fatal diseases in children in Guinea Bissau, United Republic of Tanzania and Uganda [19,26,29], where fatal outcomes were explained by factors related to the recognition of the disease and looking for late care (Delay 1), along with delays in receiving medication and lack of equipment and drugs from health providers (Delay 3). In our study, we found that home delays are less common among those who first sought care from an APE.

In the absence of routine civil registration and vital statistics (CRVS) systems, estimates suggest that one in two deaths go unrecorded globally every year in terms of medical causes [45]. We show in our study that as many as 92% of children died away from a hospital or health facility, and without a vital events registration system at community level, these deaths would largely go uncounted. With Mozambique now at the forefront of countries scaling up a national CRVS, the APEs could potentially play an important role in notifying the CRVS registrars about vital events in their catchment population. However, with only 117 deaths recorded the by 275 APEs in the year 2015, in a population with over 60 000 children under five-year olds and under-five mortality rate of 79 per 10 000 live births, we would have expected almost 10 times the number of deaths to have occurred. In order to support the successful scale up of the CRVS in Mozambique, the training and support of APEs in registration of all births and death in their catchment areas should therefore be seen as a priority.

A main limitation of verbal and social autopsy studies is that they are purely descriptive in nature and it is therefore not possible to determine any association between care seeking patterns and death. It has also been noted that social autopsy methodology does not sufficiently shed light on the details required to understand the complexities of decision making around care seeking for serious illnesses among mothers and babies [43]. Due to this complexity, standard SA tools only capture information on the acute illness phase and the first and last providers sought [19]. Still, there is a wide breadth and depth of existing social autopsy research in the recent literature and the methodology is seen as a powerful tool with the demonstrated ability to raise awareness, provide evidence in the form of actionable data and increase motivation at all levels to take appropriate and effective actions [39,43,46]. In a recent study by Willcox et al (2018), the authors took the death inquiry approach in Mali and Uganda a step further by adding a community intervention component; by using a panel of local health care workers and community representatives to review the investigation findings, formulate recommendations to address avoidable factors and, subsequently, oversee their implementation, an under five mortality reduction of 18%-27% was observed [39]. Further research is called for to optimise the implementation model for community-based death reviews, including verbal and social autopsy and other qualitative death inquiries, and actionable community-based response mechanisms. Linking these response mechanisms to other participatory community empowerment structures, such as village health clubs or women’s groups, could potentially address not only the harmful delays in care seeking, but also be applied to strengthen social capital among families.

Another limitation of our study could be that we used the InterVA tool to provide us with the cause of death information; a software that has been praised for its automation, simplicity and cost and time saving aspects to VA analysis, but also questioned for its performance in relation to manual physician panel review and other automated methods like SmartVA and InSilicoVA [47]. However, at the time of planning this study InterVA was the only automated method that was available for use and that was fully aligned with the latest WHO VA tool. While this means that we could have misreported the cause of death in the children in our sample, the main purpose of our study was to investigate care seeking behaviour for acute illness, and cause of death was just one of many explanatory variables that we explored in our analysis.

As countries increase verbal autopsy surveillance, it is important to consider the best way to design sustainable systems for data collection [48], to also add on community-based death reviews to maximise their cost-effectiveness, and to make the method part of routine health service delivery. This could be achieved by investigating and reviewing only a sample of deaths, as many of the same issues frequently recur, and therefore the same recommendations may often be repeated [19,39]. As the tools used for automated analysis of VA data are constantly evolving and improving, and it is important that planners and researchers keep abreast with the latest recommendations that WHO publishes.

Finally, while social capital has been identified as a key determinant for health in the WHO Commission on Social Determinants of Health [49], its usefulness in the study of health outcomes has been questioned by some scholars, who argue that the social capital literature is ‘gender and power blind’ [50], that economic and social status are more important drivers to health [51], and that some conditions (like suicide) have higher rates among those most socially included [52]. While the theoretical and empirical links between social capital and health are still not resolved, it can still provide a useful framework for what constitutes health supporting environments, and gives guidance on how to achieve them [22]. Further research is needed to better understand how and why (or why not) different aspects of social capital are associated with different health outcomes, and a more detailed tool for measuring social capital should be developed that can map and mobilise social capital in local communities as a way of achieving community action for health promotion.

## CONCLUSIONS

The leading causes of death in under-fives can be detected early, promptly treated or referred by APEs. Children who die experience major delays of various kinds, mainly at home, largely due to lack of early recognition of the disease and late decision-taking for outside care seeking. Distances to APEs and to referral facilities likely contribute to these delays, both for moderately and severely sick children. Efforts to increase caregiver awareness of illness in children are urgently needed, as well as stronger referral linkages with facility providers. A review of the geographical coverage of APEs, and the implications of the 80/20 time split for preventive/promotive vs curative services, should be conducted.
